# ASPECTS Interobserver Agreement of 100 Investigators from the TENSION Study

**DOI:** 10.1007/s00062-020-00988-x

**Published:** 2021-01-27

**Authors:** Noel van Horn, Helge Kniep, Gabriel Broocks, Lukas Meyer, Fabian Flottmann, Matthias Bechstein, Julia Götz, Götz Thomalla, Martin Bendszus, Susanne Bonekamp, Johannes Alex Rolf Pfaff, Paulo Roberto Dellani, Jens Fiehler, Uta Hanning

**Affiliations:** 1 Department of Diagnostic and Interventional Neuroradiology, University Medical Center Hamburg Eppendorf, Martinistraße 52, 20246 Hamburg, Germany; 2 Department of Neurology, University Medical Center Hamburg-Eppendorf, Hamburg, Germany; 3 Department of Neuroradiology, University of Heidelberg, Heidelberg, Germany; 4 Forschungszentrum Jülich, Institute of Neurosciences and Medicine, Jülich, Germany

**Keywords:** Acute stroke therapy, Brain, Endovascular treatment, Interrater reliability, Ischemic stroke, Krippendorff’s α

## Abstract

**Purpose:**

Evaluating the extent of cerebral ischemic infarction is essential for treatment decisions and assessment of possible complications in patients with acute ischemic stroke. Patients are often triaged according to image-based early signs of infarction, defined by Alberta Stroke Program Early CT Score (ASPECTS). Our aim was to evaluate interrater reliability in a large group of readers.

**Methods:**

We retrospectively analyzed 100 investigators who independently evaluated 20 non-contrast computed tomography (NCCT) scans as part of their qualification program for the TENSION study. Test cases were chosen by four neuroradiologists who had previously scored NCCT scans with ASPECTS between 0 and 8 and high interrater agreement. Percent and interrater agreements were calculated for total ASPECTS, as well as for each ASPECTS region.

**Results:**

Percent agreements for ASPECTS ratings was 28%, with interrater agreement of 0.13 (95% confidence interval, CI 0.09–0.16), at zero tolerance allowance and 66%, with interrater agreement of 0.32 (95% CI: 0.21–0.44), at tolerance allowance set by TENSION inclusion criteria. ASPECTS region with highest level of agreement was the insular cortex (percent agreement = 96%, interrater agreement = 0.96 (95% CI: 0.94–0.97)) and with lowest level of agreement the M3 region (percent agreement = 68%, interrater agreement = 0.39 [95% CI: 0.17–0.61]).

**Conclusion:**

Interrater agreement reliability for total ASPECTS and study enrollment was relatively low but seems sufficient for practical application. Individual region analysis suggests that some are particularly difficult to evaluate, with varying levels of reliability. Potential impairment of the supraganglionic region must be examined carefully, particularly with respect to the decision whether or not to perform mechanical thrombectomy.

## Introduction

Alberta Stroke Program Early CT Score (ASPECTS) is a diagnostic tool for the assessment of the extent of early ischemic lesions in anterior circulation stroke and is used to select patients for mechanical thrombectomy (MT) [1, 2]. As a semiquantitative grading system, ASPECTS estimates the extent of early ischemic changes on non-contrast computed tomography (CT) for ten regions within the middle cerebral artery territory and has been demonstrated to be predictive of clinical outcome [3].

Clinical guidelines on the management of acute ischemic stroke (AIS) patients with large vessel occlusion recognize a baseline ASPECTS ≥ 6 as a key selection criterion for MT [4, 5]; however, different ASPECTS thresholds have been employed to selectively enrol patients in various clinical trials, thereby complicating stroke assessment and subsequent therapeutic considerations in the clinical routine [6–11]. Of these trials two (MR CLEAN and THRACE) independently demonstrated the benefit of MT based on non-contrast computed tomography (NCCT), the modality most commonly used for initial evaluation [4, 10, 11]. Although ASPECTS has also been shown to be useful in identifying patients eligible for MT who present in the late time window, selection of this subgroup is typically based on advanced imaging criteria [5, 12–15].

The NCCT is an essential diagnostic tool in the management of AIS patients due to its speed and broad availability as well as its accuracy in excluding intracranial hemorrhage; however, the level of interrater agreement in the interpretation of early ischemic lesions on NCCT varies in the literature [16, 17] and is limited, reported by a systematic review analyzing 15 studies on interrater reliability ranging from fair to substantial agreement according to the Fleiss classification [18]. Despite the importance of this diagnostic tool in the selection of patients for MT, no study examining the interrater reliability among a large number of readers has been performed. We hypothesized that the interrater reliability decreases with an increasing number of readers, regardless of expertise or experience.

With the efficacy and safety of thrombectomy in stroke with extended lesion and extended time window (TENSION) trial [19], we sought to evaluate the interrater reliability of a large number of readers in the assessment of total baseline ASPECTS, as well as the individual ASPECTS regions, in patients with severe anterior circulation stroke (ASPECTS 3–5). Our goal was to identify how such potential rater-dependent differences affect the treatment decision-making process.

## Methods

This retrospective study was approved by the leading ethics committee of the local ethics committee. All patient data, study protocols and procedures were conducted in accordance with the Declaration of Helsinki and informed consent was obtained. All data used for training and validation in this study are available from the corresponding author upon reasonable request.

### TENSION—Study Design

The TENSION trial is a European investigator-initiated, prospective, open label, blinded endpoint (PROBE) [19], randomized, controlled, two-arm trial to examine the safety and effectiveness of MT compared to best medical care alone in the treatment of AIS patients with extensive early ischemic lesions (defined by an ASPECTS of 3–5) in an extended time window (up to 12 h from onset or last seen well) [20]. Only patients presenting with AIS due to focal occlusion of the M1 segment of the MCA, and/or the intracranial segment of the distal internal carotid artery, determined by either magnetic resonance angiography or computed tomography angiography were included. Subjects who met the inclusion criteria were randomized in a 1:1 ratio to either (1) best medical care alone or (2) best medical care with MT. The primary endpoint was functional outcome after 90 days post-stroke measured by modified Rankin scale (mRS90).

### TENSION—ASPECTS Image Reading Academy

The TENSION ASPECTS Image Reading Academy, a web-based training tool, was developed to qualify investigators for study-specific image assessment and ASPECTS reading (Eppdata, Hamburg, Germany). To that end, 56 cases from our stroke database were identified with a preliminary ASPECTS of 0–8, and evaluated by 4 highly experienced neuroradiologists (> 20 years of experience each, GOLD rater) in accordance with the ASPECTS grading system. The image assessment tool allowed the evaluator to window, zoom, and pan individually at a slice thickness of 4 mm. For 30 of the cases at least 3 of the 4 GOLD raters were in agreement for each individual ASPECTS region. Of these, 10 were implemented in the online training tool as training module cases, while the remaining 20 comprised the rating module. Only those who successfully completed the training modules (at least 80% correct answers) were granted participation in rating module and subsequently included for further analysis.

### ASPECTS Scoring

The first 100 TENSION investigators who successfully concluded the above defined ASPECTS training and testing were analyzed. All of them were experienced radiologists whose everyday clinical practice is to select AIS patients for MT. Early ischemic change was defined as the presence of hypodensity and/or loss of grey-white differentiation. Raters were not blinded within the test module to clinical details, affected site, time since onset, age and NIHSS. Raters provided a score for each ASPECTS region in a binary manner with 1, scoring for an affected region, and 0 for an unaffected one. Total ASPECTS score of 0–10 was simultaneously calculated based on these per region ratings and displayed to the raters.

### Statistical Analysis

Concordance of ASPECTS was evaluated using percent and interrater agreement (Krippendorff’s α [21]) at two different levels of tolerance allowance: a) zero tolerance (because deviations of one scoring point could lead to different therapeutic decisions) and b) tolerance ranges according to the TENSION inclusion criteria (ASPECTS 0–2 vs. 3–5 vs. 6–10). Concordance for binary per region ratings were calculated for percent agreement and interrater agreement using Gwet’s AC1 [22]. These metrics have been found to be unbiased in cases of high prevalence [21] (e.g., infarct demarcation in the insular region is present in almost all AIS patient scans). Values of interrater agreement are demonstrated with 95% confidence intervals (bootstrap using 1000 replicates). Furthermore, percentage shares of concordant ratings were calculated per region and per case (all raters with concordant ratings: 100%; 50/50 scoring: 50%). All data analyses were conducted using R 3.6.2 (R Core Team, 2019, Vienna, Austria) with irrCAC 1.0 and irr 0.84.1 packages [23].

## Results

**Fig. 1:**
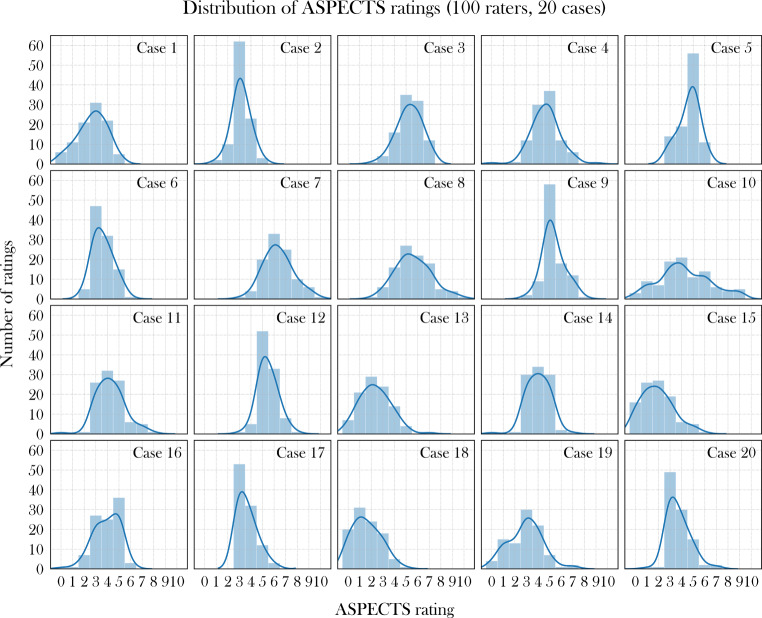
Frequency distribution of Alberta Stroke Program Early CT Score (ASPECTS) ratings for each of the 20 cases

Total ASPECTS rating frequency distributions per case were visualized using histogram plots (Fig. 1). The percent agreement for total ASPECTS ratings at zero tolerance allowance was 28%, with an interrater agreement of 0.13 (Krippendorff’s α; 95% CI: 0.09–0.16; *p*-value < 0.01), suggesting low agreement between raters. When calculated using the tolerance allowance according to TENSION inclusion criteria ranges (ASPECTS 0–2 vs. 3–5 vs. 6–10), percent agreement was 66%, with an interrater agreement of 0.32 (Krippendorff’s α; 95% CI: 0.21–0.44; *p*-value < 0.01).

**Fig. 2:**
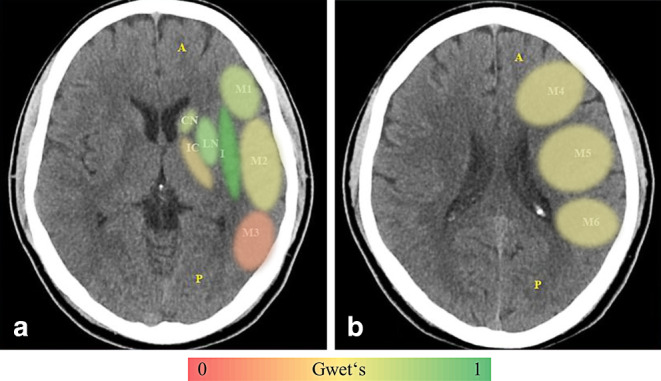
Illustrates ASPECTS per region agreement on the level of the basal ganglia (**a**) and immediately above (**b**) calculated using Gwet’s AC1 on NCCT with low (*red*), moderate (*yellow*) and high (*green*) agreement. *I* insula, *IC* internal capsule, *C* caput, *L* lentiform, *M1–M6* media cerebral artery territory M1–M6, *ASPECTS* Alberta Stroke Program Early CT Score, *NCCT* non-contrast computed tomography

**Fig. 3:**
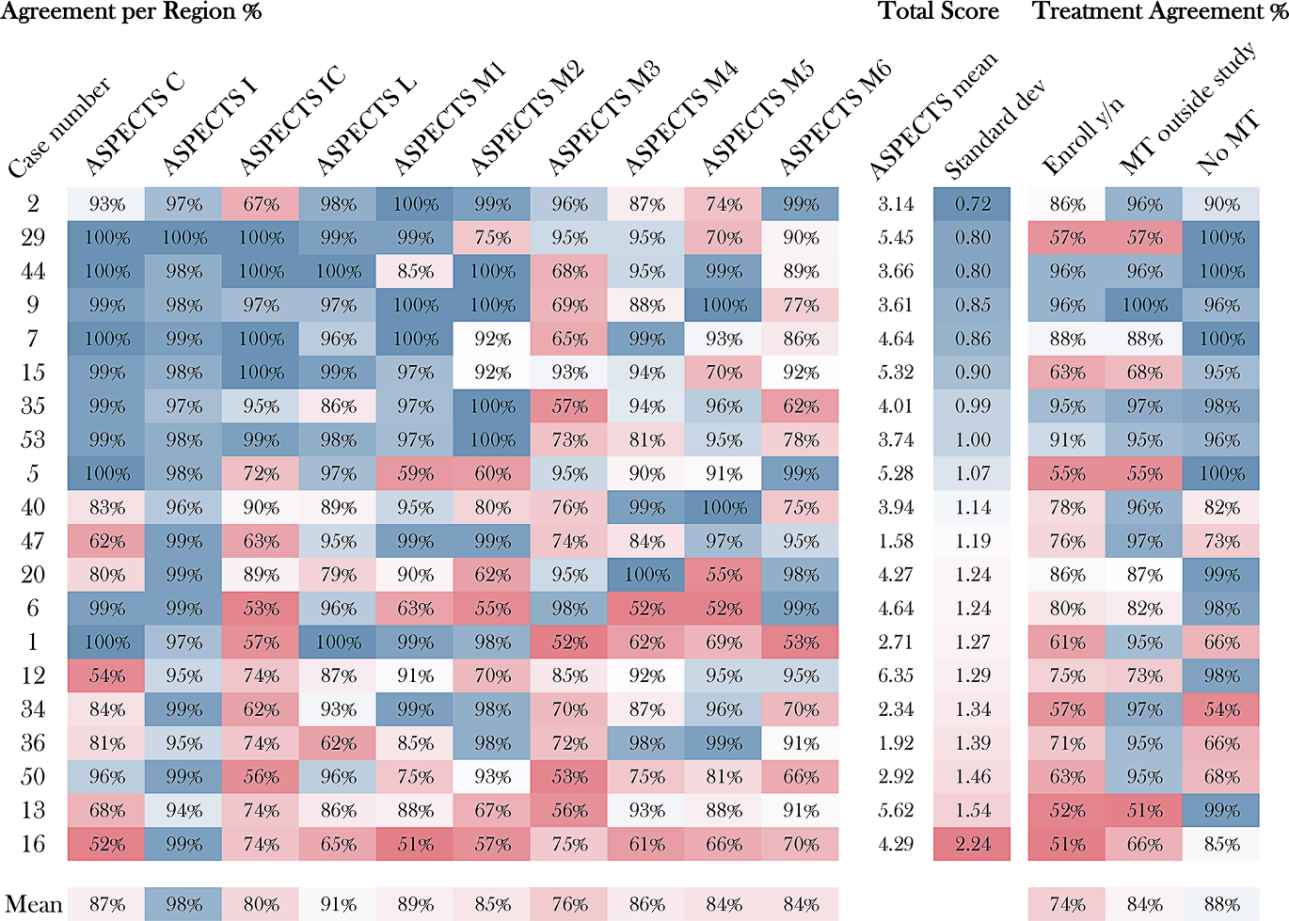
Demonstrates rating concordance among all raters regarding ASPECTS rating of all 20 cases for each ASPECTS region, mean concordance for all cases and concordance for enrolment decision for mechanical thrombectomy. Mean ASPECTS is illustrated according to ASPECTS with standard deviation (standard dev) sorted by lowest to highest standard dev (*blue*, high concordance; *red*, low concordance). *I* insula, *IC* internal capsule, *C* caput, *L* lentiform, *M1–M6* media cerebral artery territory M1–M6, *ASPECTS* Alberta Stroke Program Early CT Score

The percent and interrater agreement of the individual ASPECTS regions was highest in the insular cortex, with a percent agreement of 96% and an interrater agreement of 0.96 (Gwet’s AC1; 95% CI: 0.94–0.97; *p*-value < 0.01) (Fig. 2). The lowest per region agreement was found in the M3 region, with a percent agreement of 68% and an interrater agreement of 0.39 (Gwet’s AC1; 95% CI: 0.17–0.61; *p*-value < 0.01) (Table 1). The total and individual region shares of concordant ratings are demonstrated in Fig. 3. In all cases, the highest level of concordant ratings was observed for the insular cortex, with a mean share of 98%, while the lowest was seen in the M3 region (76%).

**Table 1:** ASPECTS interrater agreement of 100 radiologists

	Percent agreement (%)	Interrater reliability	95% CI	*p*-value
*Total ASPECTS*				
Score number	28	0.13	0.09–0.16	< 0.01
Inclusion criteria	66	0.32	0.21–0.44	< 0.01
*Rating per region*				
ASPECTS C	83	0.67	0.48–0.86	< 0.01
ASPECTS I	96	0.96	0.94–0.97	< 0.01
ASPECTS IC	73	0.58	0.37–0.79	< 0.01
ASPECTS L	86	0.73	0.59–0.86	< 0.01
ASPECTS M1	84	0.76	0.59–0.93	< 0.01
ASPECTS M2	80	0.73	0.55–0.90	< 0.01
ASPECTS M3	68	0.39	0.17–0.61	< 0.01
ASPECTS M4	80	0.66	0.49–0.83	< 0.01
ASPECTS M5	78	0.62	0.42–0.82	< 0.01
ASPECTS M6	77	0.55	0.35–0.74	< 0.01

Table 1 shows percent and interrater reliability with *p*-values. Total ASPECTS was evaluated for score numbers (zero tolerance) and agreement according to TENSION inclusion criteria cut-offs (ASPECTS 0–2; 3–5; 6–10). Interrater reliability for total ASPECTS was calculated using Krippendorff’s α; binary rating per region based on Gwet’s AC1

*I* insula, *IC* internal capsule, *C* caput, *L* lentiform, *M1–M6* media cerebral artery territory M1–M6

Baseline demographic and clinical information for the participating cases showed a median age of 65.5 years (95% CI: 52.8–76.3 years) and a median NIHSS of 16 (95% CI: 14–22), with a mean large vessel occlusion distribution located 85% in middle cerebral artery, 25% in internal carotid artery, and 5% in the anterior cerebral artery.

## Discussion

Interrater agreement differs remarkably for both total ASPECTS and the individual ASPECTS regions [24]. In our dataset, a wide frequency distribution for total ASPECTS evaluation was found. When readings of the individual regions were compared, the insular cortex showed the highest agreement, whereas the M3 region showed the lowest. This is an important finding, as ASPECTS is a well-established and frequently used diagnostic tool for the selection of patients for MT [4, 5]. The reasons for disagreement among readers, as well as the limitations of this selection tool, should therefore be clarified.

Varying ASPECTS thresholds are used to identify suitable patients for MT [6–11]. The 2019 updated “Guidelines for the Early Management of Patients with Acute Ischemic Stroke” recommend an ASPECTS threshold of 6 [4]; patients with a lower score are unlikely to experience good outcome [5]. In contrast, a recently conducted meta-analysis pooling 17 studies reported that these patients may benefit from successful reperfusion, with a higher probability of functional independence after 90 days and no greater risk of symptomatic intracranial hemorrhage [25]. Equal consideration should be given to both image evaluation and the patient’s clinical condition in the decision for best possible treatment; however, especially in patients with low ASPECTS (i.e., < 6), there seems to be some controversy over which imaging and clinical factors most affect clinical outcome. Indeed, most centers make therapeutic strategy decisions on a case by case basis.

Since first introduced in 2000 by Barber et al., varying ASPECTS thresholds have been implemented [1]. In the first subsequent study conducted by the Calgary group, a high level of disagreement among six physicians (three non-neuroradiologists and three neuroradiologists) was reported [26]. A further study by Gupta et al. [16] reported moderate interrater agreement with a κ = 0.53 (Cohen’s κ) between 2 experienced neuroradiologists for 155 patients, while Finlayson et al. [27] demonstrated very good agreement among 4 readers (2 neuroradiologists and 2 neurologists) for 181 patients using Cronbach’s α accounting for internal consistency, with an α = 0.83. Yet another recently conducted study by Nicholson et al. [24] found that overall agreement was relatively good, with a κ = 0.66 (Cohen’s κ) at an ASPECTS threshold of 5.

To date, most studies examining interrater agreement have been heterogeneous and have never examined more than six readers, making comparability to our study difficult [16, 27–29]. In order to analyze 100 experienced readers for total ASPECTS, Krippendorff’s α was used as a measure for interrater agreement [30] and showed only slight agreement. The frequency distribution of the total ASPECTS ratings also varied among the individual cases. Widest distribution was observed for case number 10, one of the oldest (78 years) and most severely affected (NIHSS of 21) cases with two occluded vessels (middle cerebral artery and internal carotid artery), which in this constellation may have had a negative impact on image evaluation.

In line with our findings, Nicholson et al. [24] reported the highest level of agreement for the insular cortex, with a κ = 0.56 (Cohen’s κ), the lowest being observed in the M3 (κ = 0.34) and internal capsule (κ = 0.44) regions. Similarly, the four observers in the study conducted by Finlayson et al. [27] had the highest level of concordance for the lentiform nucleus, with a Cronbach’s α of 0.82, and the lowest for the internal capsule (α = 0.41).

Both anatomical and methodological factors could explain why the agreement among different ASPECTS territories is so varied. In general, the basal ganglia are well-defined and rather easy to distinguish due to the grey-white contrast provided by the attenuation of the surrounding structures on NCCT. The high level of agreement for the insular cortex might be due to the contrast provided laterally by the liquor-isodense Sylvian fissure and bordering brain parenchyma of the operculum and medially by the relatively hypoattenuated external capsule. Furthermore, vulnerability to ischemic changes due to hypoperfusion varies between different locations of the brain, with the insular cortex, precentral gyrus and basal ganglia being the most sensitive [31]. On the contrary, the internal capsule is by nature distinguishable due its basic hypoattenuation compared to surrounding structures in NCCT; however, further hypoattenuation in the case of infarction could be overlooked. Moreover, if the surrounding caudate and lentiform nuclei also undergo loss of attenuation, the internal capsule may become more difficult to define, particularly if they are affected at different rates, despite a shared blood supply.

In the subganglionic and supraganglionic nuclei territories (M1–M6), differentiation of the cortex is a main criterion for early infarct detection. In order to provide a realistic diagnostic scenario, the training tool used in the study enables all raters to individually adjust window width and center for every single NCCT evaluated. Due to their location bordering the skull, beam hardening artifacts can make differentiation of these locations more difficult. Another reason the M3 region may have such a low level of agreement could be due to its relatively infrequent occurrence [32]. These territories can be of high functional eloquence. Excluding patients from MT based on involvement of these regions should therefore be carefully considered, particularly in the light of the limited interrater reliability of these locations.

We believe that this study makes an important contribution to the field of AIS therapy because it shows that despite its widespread application and acceptance, the ASPECTS grading tool is not without flaws. This is underlined by the high degree of interrater variability in both total ASPECTS scoring and in the evaluation of the individual ASPECTS regions among a large number of experienced neuroradiologists. At the zero tolerance level, the total ASPECTS scoring interrater agreement reliability was rather low. At a tolerance level according to the TENSION inclusion criteria (0–2, 3–5, 6–10), percent agreement (66%) seems sufficient for clinical routine and for study enrolment but did not meet the criteria from a strictly statistical point of view: however, responses for patient enrolment are not evenly balanced with extreme prevalence in most cases. This imbalance leads to a very low expected disagreement (which is influenced by the prevalence) and results in low reliability statistics (as the observed disagreement is higher). In both settings of a clinical trial and the everyday routine, a one-point scoring deviation may determine whether a patient suffering from AIS undergoes MT or best medical treatment alone. Moreover, with the knowledge gained from an increasing number of studies, more and more limitations to ASPECTS are being unmasked. For example, the score lacks clearly defined territory borders, unequally weights different territories, and is limited to the anterior circulation [33]. Perhaps different levels of sub-scoring for each individual region could further improve ASPECTS ratings.

When ASPECTS is applied to advanced imaging, interrater reliability improves. For example, ASPECTS evaluated on computed tomography perfusion [27] and diffusion weighted magnetic resonance imaging [34], which are based on physiological and hemodynamic changes, is more sensitive compared to NCCT-ASPECTS for the assessment of imaging abnormalities. Furthermore, automated software and deep learning algorithms are currently being tested on computed tomography perfusion-based imaging protocols to maintain or even improve interrater reliability, including that among non-neuroradiologists [35]. Preliminary results show that automated computed tomography perfusion of AIS patients in the emergency setting tends to overestimate the infarct core, as confirmed by follow-up CT after 24 h [36].

One major limitation of our study is the patient population. The observed rating results are probably poorer than in real life because of the unusually low ASPECTS population average compared to typical stroke patients. This likely leads to an inherently higher probability of disagreement. This was in part corrected for by the study inclusion selection criteria, which were based on a relatively high agreement among the four GOLD raters. Moreover, the ASPECTS range of 3–5 and the need for decision making to enrol the patient into the TENSION study could have influenced the observers. Lastly, readings were typically done on regular computer monitors, with the software running on different web browsers and operating systems, likely under suboptimal environmental/lighting conditions.

## Conclusion

Interrater agreement reliability for total ASPECTS and study enrollment was relatively low but seems sufficient for practical application. Per region analysis suggests that some ASPECTS regions are particularly difficult to evaluate, with varying levels of interrater reliability. Based on our observations, a decision to exclude patients from MT based on involvement of the supraganglionic regions should be considered with particular care. We consider these observations to be practically relevant as they are based on the findings of a large number of raters, all with considerable job experience and who are all highly motivated to achieve good rating results and, more importantly, the best patient outcome.
